# Prediction of Myopia in Adolescents through Machine Learning Methods

**DOI:** 10.3390/ijerph17020463

**Published:** 2020-01-10

**Authors:** Xu Yang, Guo Chen, Yunchong Qian, Yuhan Wang, Yisong Zhai, Debao Fan, Yang Xu

**Affiliations:** School of Computer Science and Technology, Beijing Institute of Technology, Beijing 100081, China; 2220170628@bit.edu.cn (G.C.); 3220180844@bit.edu.cn (Y.Q.); 3220180872@bit.edu.cn (Y.W.); 3220180899@bit.edu.cn (Y.Z.); 3220180792@bit.edu.cn (D.F.); 3220180886@bit.edu.cn (Y.X.)

**Keywords:** machine learning, myopia in adolescents, correlation analysis, artificial intelligence

## Abstract

According to literature, myopia has become the second most common eye disease in China, and the incidence of myopia is increasing year by year, and showing a trend of younger age. Previous researches have shown that the occurrence of myopia is mainly determined by poor eye habits, including reading and writing posture, eye length, and so on, and parents’ heredity. In order to better prevent myopia in adolescents, this paper studies the influence of related factors on myopia incidence in adolescents based on machine learning method. A feature selection method based on both univariate correlation analysis and multivariate correlation analysis is used to better construct a feature sub-set for model training. A method based on GBRT is provided to help fill in missing items in the original data. The prediction model is built based on SVM model. Data transformation has been used to improve the prediction accuracy. Results show that our method could achieve reasonable performance and accuracy.

## 1. Introduction

With the growth of social consumption and the increasing popularity of hand-held electronic terminal equipment, the incidence of myopia continues to rise. According to literature, myopia has become the second most common eye disease in China, and the incidence of myopia is increasing year by year, and showing a trend of younger age. According to incomplete statistics, only 10% or 20% of people in China developed myopia in the 1960s. However, according to a new World Health Organization study, the number of myopic patients in China now is almost half of the total population [1].

Because of the heavy burden of schoolwork and the growth and development of the eyes, bad eye habits and excessive eye use can easily lead to myopia in adolescents. Myopia has become the primary problem that puzzles the development of adolescents in China. Teenagers are the hardest hit areas of myopia. The myopia rate of senior high school students and college students has exceeded 70%, and the myopia rate of teenagers ranks first in the world. The myopia rate of primary school students in China has increased from about 10% in the first grade to nearly 40% in the sixth grade. And the myopia rate of primary and middle school students in the United States is also 10%.

The large base of Chinese population, combined with the high prevalence of myopia, requires that attention be paid to the prevention of myopia, especially for adolescents.

For the prevention of myopia, the traditional research methods in China perform not so well. Previously, we prevent myopia in adolescents mostly based on experiences or parents’ instinct. The effect is not good. In this paper, we want to build a prediction model, and we want to know which factors have more great impact on myopia, thus to help us to formulate policy to propagandize or explain these to adolescents’ parents, to enhance the myopia prevention. With the advent of big data era, the means of data mining and machine learning are becoming more and more developed. In this paper, we try to analyze the related factors affecting the incidence of myopia in adolescents combined with machine learning technology, in order to put forward the corresponding prevention and treatment strategies.

The followings are organized as: Section 2 discusses about related works; Data preprocessing method is described in Section 3; Our prediction model is introduced in Section 4; Results is given in Section 5; Conclusion is given in Section 6.

## 2. Related Works

Li et al. [2] have presented an evaluation scheme which combines K-means with weighted least squares to analyze lens fitting. They assume the treatment zone of OrthoK lenses is an ellipse, and K-means is used to cluster the data points, and weights of each point are assigned based on the results to increase accuracy. Then, the parameters of ellipse can be calculated by weighted least squares based on the weights.

Kaya et al. [3] have presented a work aimed to classify the individuals who have hypermetropia and myopia refractive disorders or not. They used horizontal and vertical Electrooculogram (EOG) signal data from the right and left eyes of the individuals. The performance of the data was investigated by using Logistic Regression (LR), Naive Bayes (NB), Random Forest (RF) and REP Tree (RT) data mining methods.

Xu et al. [4] have provided a unified Multiple Kernel Learning framework called MKL to detect ocular diseases, based on the existence of multiple informatics domains. Their framework is capable to learn a robust predictive model by effectively integrating discriminative knowledge from different informatics domains and incorporating pre-learned Support Vector Machine (SVM) classifiers simultaneously.

Bu et al. [5] have presented a causal inference methods to estimate the treatment effects of high cholesterol on myopia. They first estimate the average treatment effect for treated group (high cholesterol participants) on myopia by conducting two causal inference methods. Then verify the estimation by performing pseudo treatment and sensitivity analysis.

## 3. Data Preprocessing

The purpose for data preprocessing is to improve the quality of data, to enhance the prediction accuracy. In this work, data preprocessing process mainly includes: (1) Factor correlation analysis; (2) Feature selection; and (3) Fill in the missing items.

### 3.1. Data Description

The data used in this paper is collected from several primary school of Henan province of China. Data are collected form students in grade 1 to grade 6, including measured data of students’ eye and their behavior data. Data are collected for 3112 students. The proportion of male students is 57.8%. However, after an investigation of the original data, we found out that although the data for students in grade 1 is complete, there are a lot of missing items in data for students in grade 2 to grade 6, especially the diopter data (RA). The missing rate of each sample factor is about 15%, the highest is 40%.

In this paper, we aim to build a myopia prediction model based on big data concept. The goal is to build a model could predict the myopia situation of grade 6 students based on the evaluation of their measured data and behavior data from grade 1 to grade 5.

There are in total 200 factors in the original data. These factors cover students’ individual activity, their own eye condition, parental heredity, individual physiology, eye habits, environment, diet and so on.

We have conducted data cleaning, and data integration, before we move on to correlation analysis. Data cleaning mainly deals with all kinds of anomalies in the original data. In this paper, we mainly delete some unreasonable outliers according to the investigation of myopic literature. In order to study the visual development of normal adolescents, a few samples of congenital amblyopia and a few samples with defects in the eyes were also deleted. Data integration is to summarize and assemble the data in different data sources into the same data store for unified storage and management. The CSV file is a common data summary file for medical data, where we integrate all the data into the same CSV file so that we can analyze and process. In addition, we have made continuous value discretization, dualization, normalization and so on to the original data so as to obtain valid data that can be processed in a later time.

### 3.2. Factor Correlation Analysis

In medical research, factor correlation analysis can usually be used to verify the linear relationship between two variables. The significance test is an important criterion to judge whether the medical statistical results are significant. When the *p* value under the assumption is less than the significant level we set, we can think that the calculated results are significant, and if the *p* value is greater than the significant level, the statistical results may not be significant.

The results of the univariate correlation analysis method are shown in Table 1.

According to results, a lot of factors have *p* value smaller than 0.05, which means they are significant in statistical. The results of multivariate analysis based on multivariate linear regression method are shown in Table 2.

According to results, correlation values of factors like DAI, GENDER, JG, YW, AL, K1, K2, WHIM, and EGG are all negative, and *p* values are all lower than 0.05; while correlation values of factors as JTR, YTR, PULSE, COLA, and REDM are all positive, and with *p* values lower than 0.05.

### 3.3. Feature Selection

In the original data, there are a lot of factors. The purpose of feature selection is to find a subset of original factors which could be used to approximately represent the original data-set while has a rather small scale.

There are a lot of feature selection methods. In this paper, feature selection is done based on univariate correlation analysis and multivariate correlation analysis. The flow of this method is:

1. Calculate the correlation value and *p* value of each factor through univariate correlation analysis;
2. Construct preliminary feature sub-set by selecting factors having *p* value smaller than the pre-set significant level;
3. Build multivariate regression model with preliminary feature sub-set, and perform multivariate correlation analysis;
4. Decide the final feature sub-set according to the result of multivariate correlation analysis.

**Figure 1:**
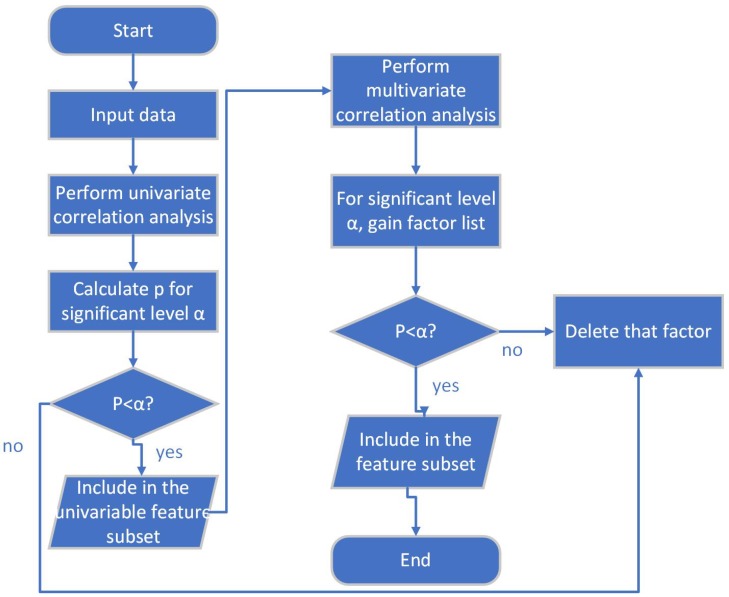
Flow of the feature selection method.

The flow of this method is shown in Figure 1.

According to the result, the final feature sub-set is constructed as a collection of factors: DAI, GENDER, JG, YW, AL, K1, K2, WHIM, EGG, JTR, YTR, PULSE, COLA, REDM, and RA. RA represents the Diopter data. Because diopter determines the visual development of adolescents and is a key variable in myopia prediction, it has also been included in the feature sub-set in this paper.

### 3.4. Fill in the Missing Items

The data used in this paper are collected from grades 1 to 6 in a primary school in Henan Province. During our investigation, we found out that many diopter data are missing, especially for senior students. In order to improve the data usage, we need to fill in the missing items.

In this paper, we presented a method based on Gradient Boosting Regression Tree (GBRT) to build a regression model to fill in the missing items.

#### 3.4.1. Standard GBRT Flow

For a giving training data-set $T={\left\{\left(x_{i},y_{i}\right)\right\}}_{i=1}^{N}$, we want to find out a function f(x) to minimize the loss function L(y,f(x)), the standard flow of GBRT is:

1. Initialize the learner, and build a tree with only root node;
  (1) $$f_{0}\left(x\right)=\underset{c}{\arg\min}\sum_{i=1}^{N}L\left(y_{i},c\right)$$
2. For round index m=1,2,…,M:
  ① For i=1,2,…,N, calculate the residual:
    (2) $$r_{im}=-{\left[\frac{\partial L(y_{i},f\left(x_{i}\right))}{\partial f(x_{i})}\right]}_{f\left(x\right)=f_{m-1}\left(x\right)}$$
  ② Use residual values $r_{im}$ to fit a new regression tree, and denote its leaf node region as $R_{jm},j=1,2,\ldots ,J$;
  ③ For leaf node region j=1,2,…,J, calculate the best fitting value:
    (3) $$c_{mj}=\underset{c}{\arg\min}\sum_{x_{i}\in R_{mj}}L\left(y_{i},f_{m-1}\left(x_{i}\right)+c\right)$$
  ④ Update:
    (4) $$f_{m}\left(x\right)=f_{m-1}\left(x\right)+\sum_{j=1}^{J}c_{mj}I\;\left(x\in R_{mj}\right)$$
3. Output regression tree $f_{M}\left(x\right)$;
  (5) $$f_{M}\left(x\right)=\sum_{m=1}^{M}\sum_{j=1}^{J}c_{mj}I\left(x\in R_{mj}\right)$$

#### 3.4.2. Missing Item Filling Method Based on GBRT

Although the fitting effect of GBRT method is already very strong, the filling value of diopter data needs to be within a certain acceptable error range. If the whole set of data is directly used for training, only a single model can be obtained, which can not guarantee that the result is within the acceptable error range. Therefore, we divide the data, get the training set and test set of different sizes, and then find the appropriate model according to the test index REP (Square of R and Error Proportion) designed by us, and then complete the filling of the missing data.

Here REP is calculated as:

(6) $$REP=\frac{R^{2}}{r}$$

where $R^{2}$ is fitting goodness, and *r* is absolute error rate. $R^{2}$ can be calculated as:

(7) $$R^{2}=\frac{\sum_{i=1}^{n}{\left(f\left(x_{i}\right)-\bar{x}\right)}^{2}}{\sum_{i=1}^{n}{\left(x_{i}-\bar{x}\right)}^{2}}$$

where $x=\{x_{1},x_{2},\ldots ,x_{n}\}$ are data needs to be fit, $f\left(x\right)=\{f\left(x_{1}\right),f\left(x_{2}\right),\ldots ,f\left(x_{n}\right)\}$ are the fitted value, while $\bar{x}$ is their average value. And *r* is calculated as $r=\frac{1-n}{m}$. Here *m* represents the total number of samples, and *n* is the number of samples that satisfy the condition that e(x)<y. e(x) could be calculated as $e\left(x\right)=|x-x^{\prime }|$, where *x* is the true value, while $x^{\prime }$ is the measured value. And *y* is a predefined error range.

In the regression model, fitting goodness $R^{2}$ measures the overall fitting degree of a regression equation, and the range of $R^{2}$ is [0,1]. If the value of $R^{2}$ is closer to 1, the better the fitting degree of the regression model is; the smaller the value of $R^{2}$ is, the worse the fitting effect of the model is.

The absolute error rate *r* reflects the deviation between the real value and the predicted value of a group of data, and its range is also [0,1]. If the ratio is larger, the prediction error of the whole group is larger, and if the ratio is smaller, the prediction error of the whole group is smaller.

Based on the characteristics of the two, we propose REP (Square of R and Error Proportion). The main purpose of proposing REP is to prevent the model from being trained with high fitting goodness, but the deviation between the actual predicted value and the measured value is large. On the one hand, the one-sidedness caused by a single index is avoided, and the prediction results not only ensure the error range less than the given error range, but also ensure the fitting effect of the regression model. The larger the value of REP, the better the effect of the whole group of data predicted by the regression model, on the contrary, the worse the effect.

The data filling method used in this paper is not directly optimized for the specific regression method, but more on the basis of the test results to select the model.

The complete flow of the missing item filling method used in this paper is describe below:

1. According to data status, divide the original data-set into 2 sub-sets: Subset *M*, which includes data that have missing item, and subset *D*, which includes data have none missing item;
2. Divide *D* into training set $D_{train}$ and test set $D_{test}$ according to the proportion between *p* and 1−p;
3. Build regression model based on GBRT using $D_{train}$;
4. Test the regression model with $D_{test}$, and calculate REP;
5. Adjust the value of *p*, and record the corresponding REP value of regression model;
6. Output the regression model with maximum REP;
7. Fill in the miss items of *M* with the generated regression model.

## 4. Prediction Model of Influence Factor of Myopia in Adolescents

The goal of the prediction model is whether a sixth grader will get myopia. The prediction result is a probability value. If it is larger than 50%, we treat it as a yes, otherwise a no. We use the first grade to fifth grade data to build the prediction model. Since different kinds of factors tend to have different kinds of influence on the output, we will perform data transformation process separately for different kinds of factors to better exploit them to build the model.

**Figure 2:**
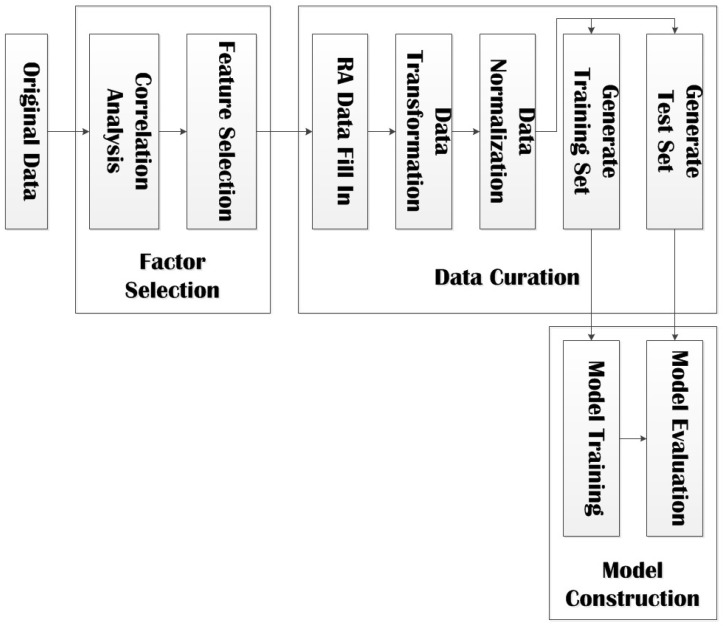
Flow of the prediction model.

The prediction model of influence factors of myopia in adolescents in this paper is built based on SVM (Support Vector Machine) method. The entire flow of our model in shown in Figure 2.

The prediction model is composed of three phases:

1. Factor Selection: Perform feature selection based on univariate correlation analysis and multivariate correlation analysis;
2. Data Curation: Fill in the missing items in RA data based on GBRT method, perform data transformation and data normalization, and divide data into training set and test set;
3. Model Construction: Train model with training set, evaluate model with test set, and output the final prediction model.

### 4.1. Data Transformation

In order to take into consideration the different influence of different kinds of factors on the prediction output, we have designed different data transformation process for different kinds of factors:

1. RA: The difference between the previous grade and the next grade of diopter data is taken to obtain the annual progress, and then the average annual progress is calculated, which is included in the feature set of model training. The average annual RA progress was used to reflect the difference of RA between individual samples;
2. JG, YW, COLA, EGG, REDM, WHIM: Data from grade 1 to grade 5 are added up and their summation is used as a feature of model training. These six variables are classified variables, involving individual activity factors and eating habits of each sample. Although the data may vary from year to year, in order to reflect the total difference of individuals in primary school, they are accumulated;
3. JTR, YTR, PULSE, AL, K1, K2: The average value of 5 grades is taken as the feature of model training. These six variables are continuous variables, describing the eye and body factors of the individual sample itself. These factors change little with time, so the average value of these factors can better reflect the eye differences between individuals;
4. DAI, GENDER: These two variables are objective variables and do not change with time, so the original value is directly used as the characteristic of inclusion model training.

### 4.2. Model Parameter for SVM

Support vector penalty coefficient and the parameters of the kernel function play an important role in learning precision and generalisation ability of regression model [6]. In SVM, the penalty coefficient is the punishment degree of the model for error. The higher the *C* setting, the more likely the model is to overfit, because the tolerance to the error becomes smaller, while the smaller the *C* setting, the more likely the model is to appear underfitting. Although the larger C can make the model perform better, the interval of the trained classification may be small, the model may not have the generalization ability, and the training time of the model will be doubled, resulting in unnecessary time waste. In disease prediction, the model with low generalization ability is not of practical significance, so we properly allow some classification errors and set the penalty coefficient to the default value of 1.0.

The kernel function in SVM is mainly divided into linear kernel function and nonlinear kernel function. Gaussian kernel function is the most commonly used nonlinear kernel function. In the nonlinear kernel function, the classification results depend on the selection of parameters, and its parameters are many, so it takes a lot of time to construct the model. The linear kernel function parameters are few, the calculation speed is fast, the model construction is fast, and can be directly used in linear divisible data. Although the dimension of our original data is high, after the feature selection the data is suitable for linearly divisible cases, and since we have carried out data transformation processing to reduce the dimension of the data, so the kernel function used in the establishment of this model is linear kernel function.

## 5. Experiment Results

### 5.1. Evaluation Metrics

The evaluation metrics used in this paper are:

1. Cross validation: This is a common metric used in model verification and evaluation. In this paper, the default 10-fold cross-validation method is used to evaluate the prediction accuracy of our model;
2. Accuracy: Accuracy represents the ratio of the number of samples with the same predicted value as the actual value to the total sample;
3. Sensitivity: Sensitivity or recall or TPR (True Positive Rate) is calculated as TPR=TP/(TP+FN), where TP represents the number of True Positive samples, while FN is the number of False Negative samples;
4. Precision: This is also called positive predictive value, is the fraction of retrieved instances that are relevant, which could be calculated as Precision=TP/(TP+FP), where FP is the number of False Positive samples;
5. f1-score: f1-score is a more combined metric, which could be calculated as f1score=2∗(precision×recall)/(precision+recall);
6. ROC Curve: ROC (Receiver Operating Characteristic) curve is another common used metric used in evaluate the prediction accuracy of a model.

### 5.2. Evaluation of Missing Item Fill

In this paper, the effect of data filling method based on GBRT is verified by experiments. Data without missing items in grade 1 and grade 2 are used as verification data-set to verify the regression filling effect under different *p* values.

**Figure 3:**
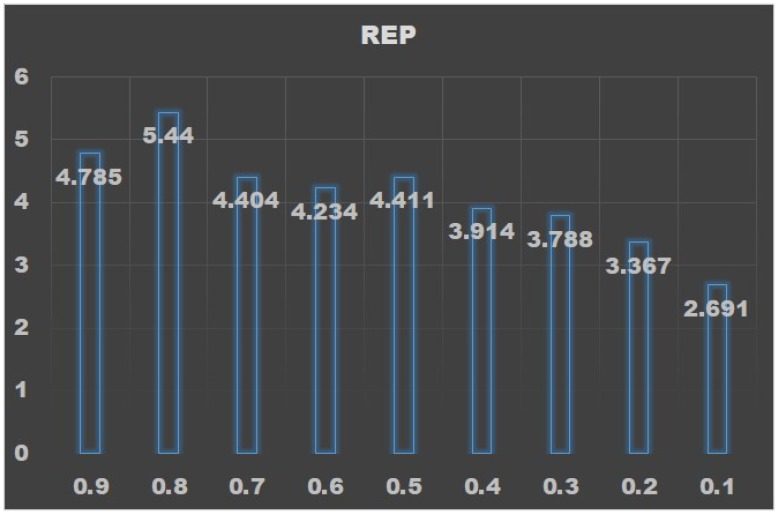
REP trend with respect to *p* value.

The results are shown in Table 3. It can be seen that, when *p* value is 0.8, *r* has the minimum value, while $R^{2}$ is approximately the maximum value. The REP trend with respect to *p* value is shown in Figure 3.

The results show that when *p* value is 0.8, REP gets the maximum value. Since when *p* value is 0.8, absolute error rate *r* also gets the minimum value, thus verifies that our method is effective.

### 5.3. Evaluation of Feature Selection

**Figure 4:**
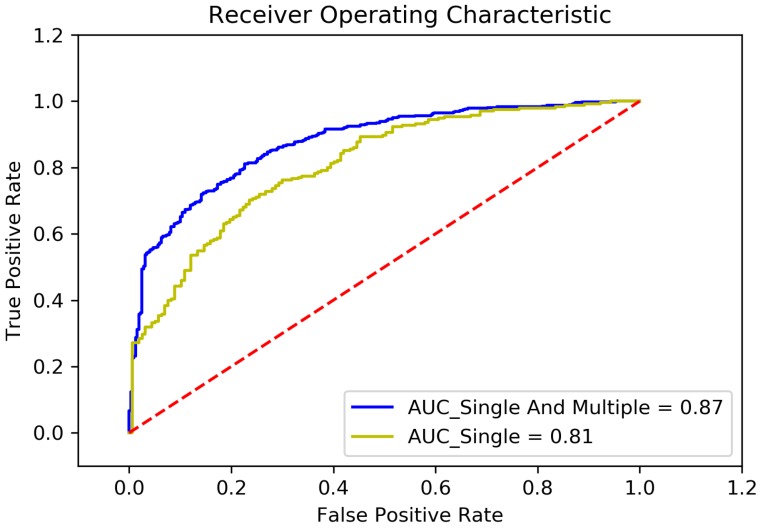
Comparison between different feature selection method.

In order to verify the effect of feature selection method used in this paper, we have conducted an experiment. The comparison result is shown in Figure 4.

The label “AUC_Single And Multiple” represents where the feature selection method based on univariate correlation analysis and multivariate correlation analysis is used. The factors listed in Section 3.3 are used to build the feature sub-set. And the label “AUC_Single” represents where the feature selection method based only on univariate correlation analysis is used. According to the result of univariate correlation analysis, 50 factors have been selected into the feature sub-set to train the model.

It could be seen from the figure that, when feature selection method based only on univariate correlation analysis is used, the accuracy of the model is 0.74, AUC area is 0.81, precision is 0.74, sensitivity is 0.86, and f1-score is 0.80; while feature selection method based on univariate correlation analysis and multivariate correlation analysis is used, the accuracy is 0.79, the AUC area is 0.87, precision is 0.79, sensitivity is 0.88, and f1-score is 0.83. According to the comparison, the feature selection method based on univariate correlation analysis and multivariate correlation analysis is more effective.

### 5.4. Evaluation of Data Transformation

In order to verify the improvement of data transformation on prediction accuracy, a comparison has been done.

**Figure 5:**
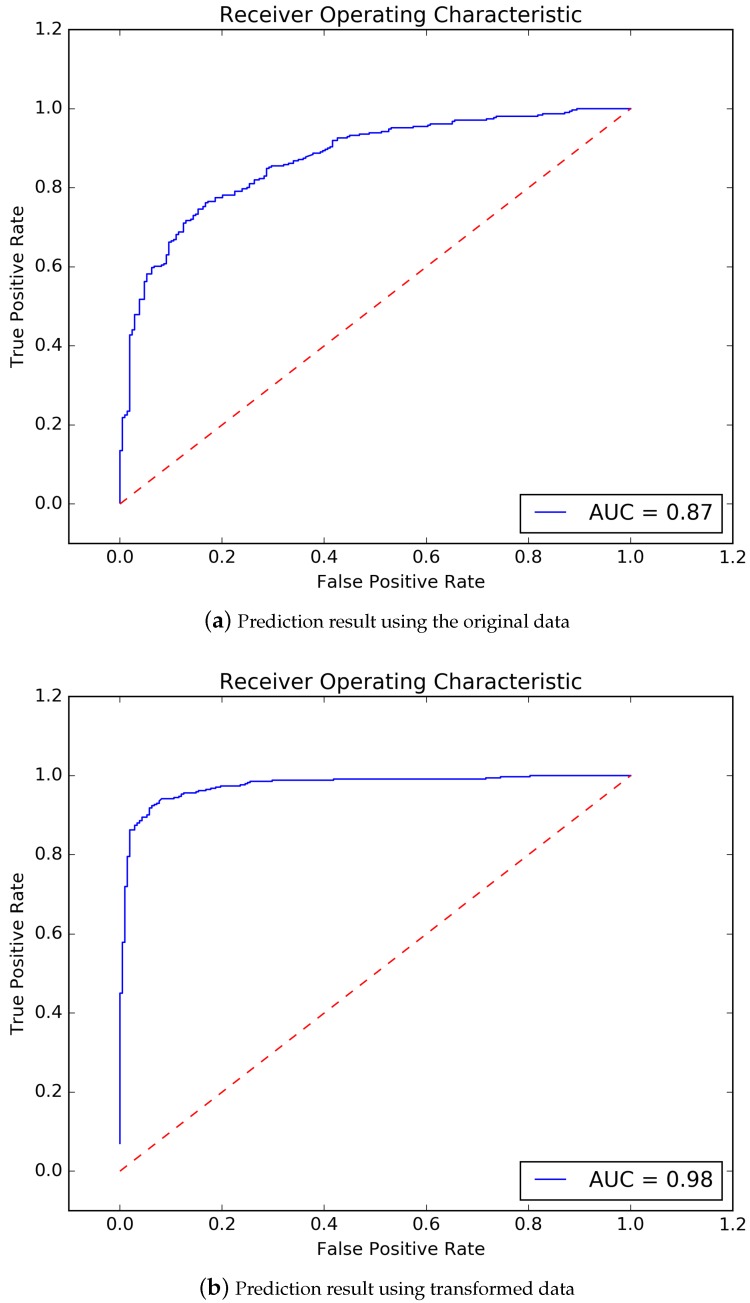
Influence of data transformation on results.

The ROC curve of using the original data to train model and predict is shown in Figure 5a. It could be seen that, the accuracy is 0.79, 10-fold cross-validation result is 0.76, precision is 0.78, sensitivity is 0.88, f1-score is 0.83, AUC area is 0.87. And the ROC curve of using the data after data transformation to train model and prediction is shown in Figure 5b. The accuracy is 0.93, 10-fold cross-validation result is 0.92, precision is 0.95, sensitivity is 0.94, f1-score is 0.94, AUC area is 0.98.

According to the comparison, we could conclude that, the prediction accuracy of the model is largely improved by performing data transformation.

### 5.5. Evaluation of Prediction Accuracy

In order to testify the performance of our method, we choose several baseline methods to compare with our method. Those baseline methods include: logistic regression, Naive Bayes, KNN, Random Forest, and BP neural network.

**Figure 6:**
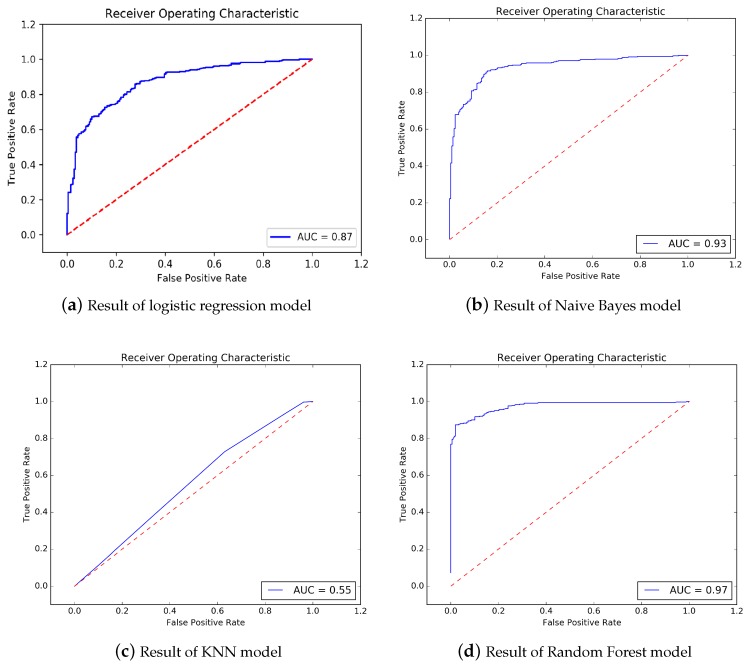

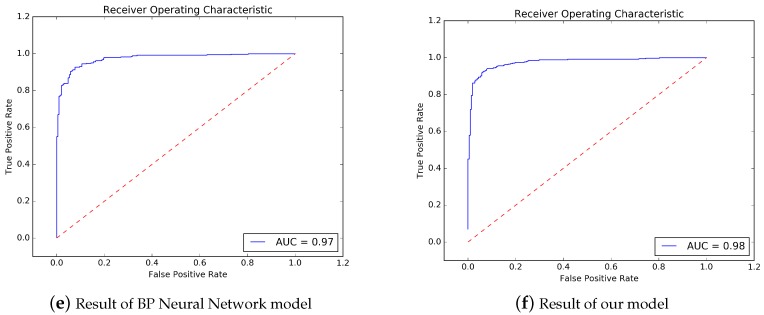
Comparison of accuracy

The prediction result of logistic regression is shown in Figure 6a. The accuracy is 0.89, 10-fold cross-validation result is 0.89, precision is 0.82, sensitivity is 0.88, f1-score is 0.85, AUC area is 0.95. The result of Naive Bayes is shown in Figure 6b. The accuracy is 0.88, 10-fold cross-validation result is 0.84, precision is 0.89, sensitivity is 0.92, f1-score is 0.90, AUC area is 0.93. The result of KNN method is shown in Figure 6c. The accuracy is 0.60, 10-fold cross-validation result is 0.58, precision is 0.66, sensitivity is 0.73, f1-score is 0.69, AUC area is 0.55. Random Forest method’s result is shown in Figure 6d. The accuracy is 0.91, 10-fold cross-validation result is 0.90, precision is 0.94, sensitivity is 0.91, f1-score is 0.92, AUC area is 0.97. BP Neural Network’s result is shown in Figure 6e. The accuracy is 0.92, 10-fold cross-validation result is 0.90, precision is 0.95, sensitivity is 0.93, f1-score is 0.94, AUC area is 0.97.

Table 4 shows the summary of comparison of our method and those baseline methods.

It could be seen that our method could provide the best Accuracy, and the best AUC compared with all other methods.

## 6. Conclusions

In this paper, a prediction model of myopia in adolescents based on both measurement and behavior data of primary school students is presented. A feature selection method based on both univariate correlation analysis and multivariate correlation analysis is used to better construct a feature sub-set for model training. A method based on GBRT is provided to help fill in missing items in RA data. The prediction model is built based on SVM model. Data transformation has been used to improve the prediction accuracy. The results show that our method could provide reasonable prediction accuracy.

Also, according to our results, we found out that some factors have positive correlation with myopia, like DAI, JG, YG, AL, K1, K2, WHIM, which we call them protective factors. While some other ones, like JTR, YTR, PULSE, COLA, REDM have negative effect on the myopia, which we call them dangerous factors.

Since our goal is to build a model which could predict the myopia in adolescents, and deduce the relation between myopia and different factors, so that we could formula policy to help preventing myopia. Future work might include more thoroughly analysis of the result and discussing of possible improvements in the methodology.

## Figures and Tables

**Table 1:** Results of univariate correlation analysis.

Factor	Description	Correlation	*p* Value
DAI	No. of parents wearing glasses	0.061	0.002
GENDER	Gender	0.067	$8.317\;\times \;10^{-4}$
RPR	Before mydriasis (right eye)	0.091	$5.991\;\times \;10^{-6}$
JTR	Close adjustment ability (right eye)	0.108	0.001
YTR	Remote adjustment ability (right eye)	0.267	$1.354\;\times \;10^{-41}$
DRNBASE	Distant vision (right eye)	0.222	$4.496\;\times \;10^{-29}$
JG	Amount of indoor activities	0.029	0.001
YW	Amount of outdoor activities	0.030	0.032
AL	Axial length	0.106	$1.546\;\times \;10^{-7}$
K1	Corneal curvature (left eye)	0.062	0.002
K2	Corneal curvature (right eye)	0.043	0.036
PULSE	Pulses per minute	0.006	0.008
TUTOR1	Participation in outdoor classes	0.282	$5.417\;\times \;10^{-42}$
TUTOR2	Participation in indoor classes	0.203	$3.620\;\times \;10^{-22}$
ETEST	If have regular eye examination	0.344	$4.732\;\times \;10^{-63}$
MSMK	Whether or not smoke	0.397	$1.826\;\times \;10^{-82}$
CELLP	Whether or not play cellphone	0.229	$6.606\;\times \;10^{-28}$
COSTM	Whether or not write with wrong posture	0.092	$1.356\;\times \;10^{-5}$
BED	Whether or not read in bed	0.261	$2.480\;\times \;10^{-36}$
COLA	Frequency of drinking carbonated drinks	0.092	$1.330\;\times \;10^{-5}$
REDM	Frequency of eating red meat	0.037	0.026
WHIM	Frequency of eating white meat	0.028	0.044
EGG	Frequency of eating eggs	0.077	$2.285\;\times \;10^{-4}$
MILK	Frequency of drinking milk	0.077	$2.672\;\times \;10^{-4}$
VOLUME	Daily amount of water drinking	0.096	$4.681\;\times \;10^{-6}$

**Table 2:** Results of multivariate correlation analysis.

Factor	Description	Correlation	Std Err	*t*	*p*
DAI	No. of parents wearing glasses	−0.0218	0.033	−0.670	0.042
GENDER	Gender	−0.0418	0.047	−0.882	0.015
RPR	Before mydriasis (right eye)	−0.0160	0.022	−0.735	0.207
JTR	Close adjustment ability (right eye)	0.0402	0.060	0.667	0.005
YTR	Remote adjustment ability (right eye)	0.2077	0.049	4.245	0.000
DRNBASE	Distant vision (right eye)	−0.4191	0.200	−2.099	0.306
JG	Amount of indoor activities	−1.043	1.13	−0.922	0.035
YW	Amount of outdoor activities	−0.0099	0.014	−0.699	0.048
AL	Axial length	−0.0420	0.073	−0.578	0.003
K1	Corneal curvature (left eye)	−0.2655	0.578	−0.459	0.046
K2	Corneal curvature (right eye)	−0.2155	0.495	−0.435	0.036
PULSE	Pulses per minute	0.0003	0.002	0.167	0.008
TUTOR1	Participation in outdoor classes	0.0871	0.047	1.869	0.062
TUTOR2	Participation in indoor classes	0.0333	0.044	0.751	0.453
ETEST	If have regular eye examination	−0.0877	0.043	−2.042	0.401
MSMK	Whether or not smoke	−0.3692	0.266	−1.387	0.166
CELLP	Whether or not play cellphone	0.0530	0.047	1.129	0.259
COSTM	Whether or not write with wrong posture	−0.0058	0.028	−0.205	0.838
BED	Whether or not read in bed	−0.0513	0.031	−1.677	0.094
COLA	Frequency of drinking carbonated drinks	0.0041	0.025	0.163	0.007
REDM	Frequency of eating red meat	0.0167	0.022	0.761	0.044
WHIM	Frequency of eating white meat	−0.0231	0.027	−0.842	0.040
EGG	Frequency of eating eggs	−0.0054	0.025	−0.214	0.013
MILK	Frequency of drinking milk	0.0163	0.031	0.532	0.595
VOLUME	Daily amount of water drinking	−0.0041	0.032	−0.130	0.897

**Table 3:** Results under different *p* value.

*p* Value	$R^{2}$	*r*
0.9	0.791	0.165
0.8	0.789	0.145
0.7	0.787	0.178
0.6	0.755	0.178
0.5	0.786	0.178
0.4	0.776	0.198
0.3	0.766	0.202
0.2	0.743	0.220
0.1	0.694	0.258

**Table 4:** Comparison of methods.

	Accuracy	10-Fold Cross-Validation	Precision	Sensitivity	f1	AUC	Specificity
Our method	93%	0.92	0.95	0.94	0.94	0.98	0.94
Logistic Regression	89%	0.89	0.82	0.88	0.85	0.95	0.88
Naive Bayes	88%	0.84	0.89	0.92	0.90	0.93	0.92
KNN	60%	0.58	0.66	0.73	0.69	0.55	0.73
Random Forest	91%	0.90	0.94	0.91	0.92	0.97	0.91
BP Neural Network	92%	0.90	0.95	0.93	0.94	0.97	0.93
